# A new species of *Heliconia* (Heliconiaceae) with pendent inflorescence, from Chucantí Private Nature Reserve, eastern Panama

**DOI:** 10.3897/phytokeys.77.11190

**Published:** 2017-02-07

**Authors:** Rodolfo Flores, Carla Black, Alicia Ibáñez

**Affiliations:** 1 Programa de Maestría en Biología Vegetal, Universidad Autónoma de Chiriquí, Republic of Panama; 2 Finca Las Chichicas, Volcán, Chiriquí, Republic of Panama; 3 Jadwin Av. 108B, Gamboa. P.O. Box 0843-00954. Balboa, Republic of Panama

**Keywords:** *Barbatae*, Cerro Chucantí, *Griggsia*, *Heliconia*, Heliconiaceae, Serranía de Majé, Zingiberales

## Abstract

*Heliconia berguidoi* (Heliconiaceae), a new species from premontane forest of eastern Panama, is described, illustrated and its conservation status evaluated. *Heliconia berguidoi* bears pink flowers, an uncommon color in this group. It differs from the Colombian species *Heliconia rhodantha* and *Heliconia sanctae-theresae*, the most similar taxa, by the combination of a petiole glabrous except for the woolly base, a very long peduncle, the perianth pubescent at the apex and staminode with cuspidate apex. *Heliconia berguidoi* is also similar to *Heliconia pogonantha* in all four of its varieties and to *Heliconia ramonensis* in two of its four varieties, but differs by a combination of the long peduncle, pink flowers and staminode with cuspidate apex. Fifty-six *Heliconia* species have been found in Panama, eighteen of them endemic.

## Abstract (es)

*Heliconia berguidoi* (Heliconiaceae), una nueva especie de bosque premontano del este de Panamá, es descrita, ilustrada y su estado de conservación evaluado. *Heliconia berguidoi* tiene flores rosadas, color poco común en este grupo. Difiere de las especies de Colombia *Heliconia rhodantha* y *Heliconia sanctae-theresae*, taxones más similares, por la combinación de un pecíolo glabro aunque lanudo en la base, pedúnculo muy largo, ápice del perianto pubescente y estaminoide con el ápice cuspidado. *Heliconia berguidoi* es también similar a *Heliconia pogonantha* en sus cuatro variedades y a *Heliconia ramonensis* en dos de sus cuatro variedades, pero difiere por la combinación de su largo pedúnculo, el color rosado de las flores y el estaminoide con ápice cuspidado. En Panamá se han encontrado cincuenta y seis especies de *Heliconia*, dieciocho de ellas endémicas.

## Introduction

*Heliconia* L. is the only genus in the plant family Heliconiaceae, which is included in the order Zingiberales (Berry and Kress 1991, APG IV 2016). This family is native to tropical America (Caribbean islands, Mexico, Central America and South America) with a small number of species in the Old World tropics, distributed from Samoa, westward to the central Indonesian island of Sulawesi (Kress 1984, Kress 1990b, Berry and Kress 1991). *Heliconia* has been formally and informally divided into five subgenera: *Taeniostrobus* (Kuntze) Griggs, *Heliconia*, *Stenochlamys* Baker, *Griggsia* L.Andersson and *Heliconiopsis* (Miq.) Kress (Andersson 1985, 1992; Kress 1984, 1990a).

The total number of *Heliconia* species is still unclear, although in a recent account Ferreira de Castro et al. (2007) registered 176 for the Neotropical region and 6 in the Pacific islands, for a total of 182 species distributed in five (5) subgenera and twenty-three (23) sections. Kress and Betancur (2009) recently described one more new species from Colombia which makes a total of 183 recognized species. In Panama, 55 *Heliconia* species and infraspecific taxa have been reported and the country has the third largest number of endemics (17), after Colombia (36) and Ecuador (21) (Kress 2003, Correa et al. 2004, Ferreira de Castro et al. 2007, Tropicos 2016).

The new *Heliconia berguidoi* has been found in the premontane forests of Chucantí Private Nature Reserve, at around 800 m, in disturbed and mature forest. Seven other *Heliconia* species occur in the area: *Heliconia latispatha* Benth., *Heliconia pogonantha* Cufod., *Heliconia metallica* Planch. & Linden ex Hook., *Heliconia nutans* Woodson, *Heliconia wagneriana* Petersen, *Heliconia platystachys* Baker and *Heliconia spathocircinata* Aristeg.

**Figure 1.**
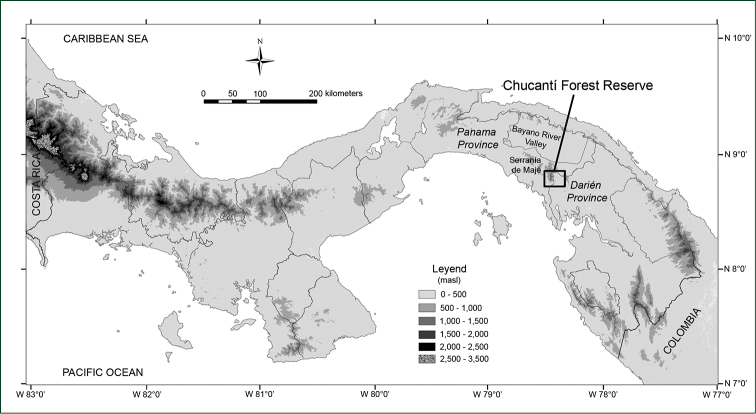
Elevation map of Panama with location of Chucantí Private Forest Reserve.

Chucantí Private Nature Reserve (404 hectares) is located on the border of Panama and Darién Provinces, on the eastern edge of Serranía de Majé, an isolated mountain range about 60 km long. It is 30 km south of the continental divide across the valley of the Bayano River and 15 km inland from the Pacific (Figure 1). The range rises gradually towards the east, with the highest point, Cerro Chucantí (1,439 m) at the eastern end (BirdLife International 2016). The reserve, which extends from around 800 m to the highest summit, harbors premontane and lower montane rain forests (Holdridge et al. 1971). According to the ecoregion classification system (WWF 2016), Chucantí is part of the Eastern Panamanian montane forests ecoregion. It has been designated an Important Bird Area (IBA) in Danger (Angehr 2003), as the extensive loss of forests due to cattle ranching activities is putting in peril the existence of several endemic bird species.

As part of a floristic inventory of the region carried out by two of the authors (Flores and Ibáñez, unpublished), ca. 250 species have been identified from more than 500 collections. At least 6 of them have been recognized as species new to science (Ortiz et al. 2016; Flores et al., submitted; Valdespino et al. in prep.), including the new *Heliconia* described here. Endemic species of fauna have also been recently described from the area (Batista et al. 2014, Bezark et al. 2013, Miranda and Bermúdez 2010). All of these novelties highlight the importance for conservation of this undercollected region of Panama. Discovering, describing and conserving biodiversity is the purpose of the *Asociación Adopta el Bosque Panamá*, owner of the Chucantí Private Nature reserve.

## Materials and methods

One live plant of *Heliconia berguidoi* was collected in Chucantí Private Nature Reserve (2006) and was grown at Finca las Chichicas (Chiriquí Province). Ten years later, a specimen from the original plant was photographed, studied under cultivation, collected, illustrated and deposited at the Herbarium of the University of Panama (PMA). Some flowers and fruits were stored in 70% ethanol and studied using a stereomicroscope.

In March 2011, a specimen of *Heliconia berguidoi* was collected in Chucantí Private Nature Reserve as part of a general floristic inventory of the area.

Each type specimen of subgenus Griggsia L.Andersson was reviewed in the JSTOR Global Plants webpage (JSTOR 2016). Some specimens of those species deposited in PMA Herbarium were also reviewed. Maps were made with the program ArcGIS version 10.1. The IUCN Red List Categories and Criteria (IUCN 2012) was used to determine the conservation status of the new species.

## Taxonomic treatment

### Heliconia berguidoi

Taxon classificationPlantaeZingiberalesHeliconiaceae

R.Flores, C.Black & A.Ibáñez sp. nov.

urn:lsid:ipni.org:names:77160178-1

Figs 2 , 3 , 4

#### Diagnosis.

This species is distinguished from other species of *Heliconia* by the combination of the long petioles (up to 180 cm), glabrous but woolly at the base; blade splitting into narrow lateral segments; peduncle red, woolly with golden hairs, very long (125-150 cm); slightly flexuous rachis; bracts spirally arranged; pink flowers, perianth pubescent at the apex and staminode with cuspidate apex.

#### Type.

PANAMÁ. Provincia de Darién: Reserva privada Chucantí, Sendero al filo (roca grande). Bosque premontano. 900 m. 8°47'33.46"N, 78°27'6.72"W, 26 agosto 2006, individuo colectado por Carla Black. Floreció en cultivo el 12 de marzo de 2016, Finca las Chichicas, corregimiento de Volcán, distrito de Bugaba, Provincia de Chiriquí. Col. R. Flores, O. Ortiz y C. Black, 3855 RF (Holotype PMA!, Isotype, MO!, SEL!, UCH!, US!).

#### Description.

**Figure 2.**
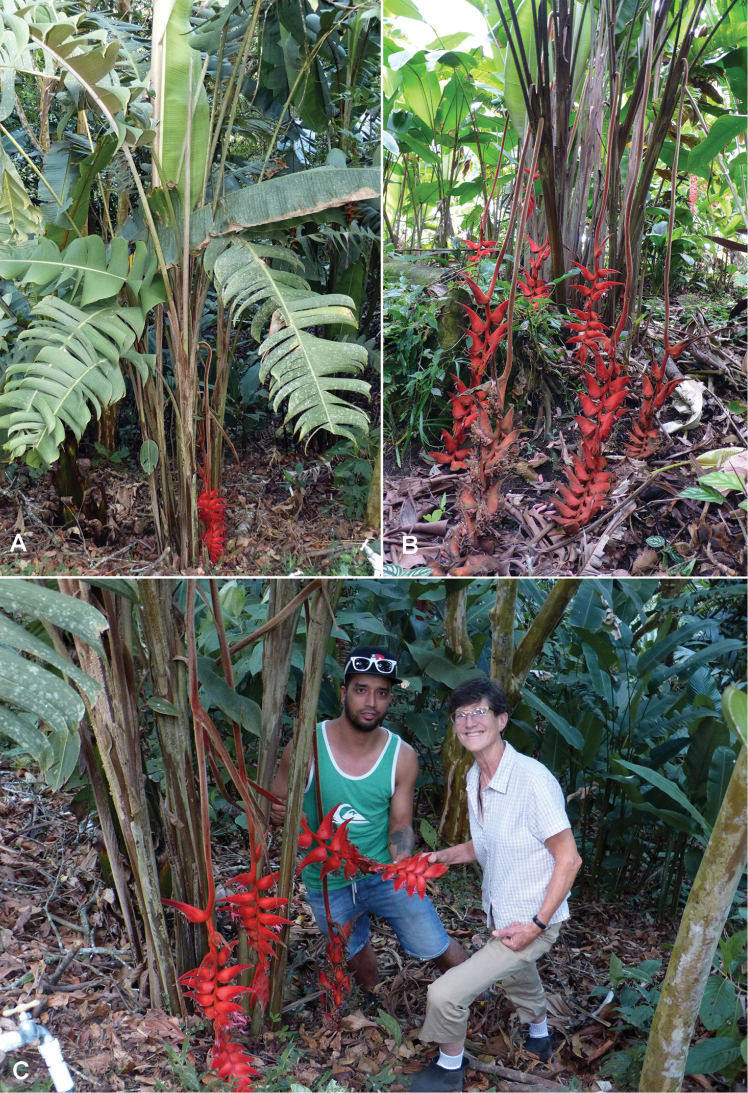
**A** Habit of *Heliconia berguidoi* **B** Inflorescences touching the ground **C** Plant with two of the authors (R. Flores and C. Black). Photos: **A**, **C** – R. Flores; **B** – C. Black.

Herb with *Musa*-like habit, 4.5–5 m tall, leafy shoots to 5 stems per group. Pseudostem green with brown lenticular spots, 160–180 cm tall, 6.5–7.5 cm in diameter; sheath glabrous but woolly on the margin. Leaves 4 per shoot, held more or less in horizontal position; petiole green, glabrous, woolly at the base, ca. 180 cm long, 2 cm in diameter; symmetrical blades splitting into narrow lateral segments with the base truncate, unequal, splitting into narrow lateral segments, apex acuminate, the upper surface green, midrib light green and glabrous, the lower surface light green, midrib green-reddish, glabrous, the largest blades up to 160 cm long and ca. 48 cm wide (Figures 2A, 4A, C).

Inflorescence pendent, up to 220 cm long; peduncle red, woolly with golden hairs, 125–150 cm long, 2 cm in diameter; rachis red, slightly flexuous, velutinous with golden hairs, 1.5 cm in diameter at the base (Figures 2, 4B).

**Figure 3.**
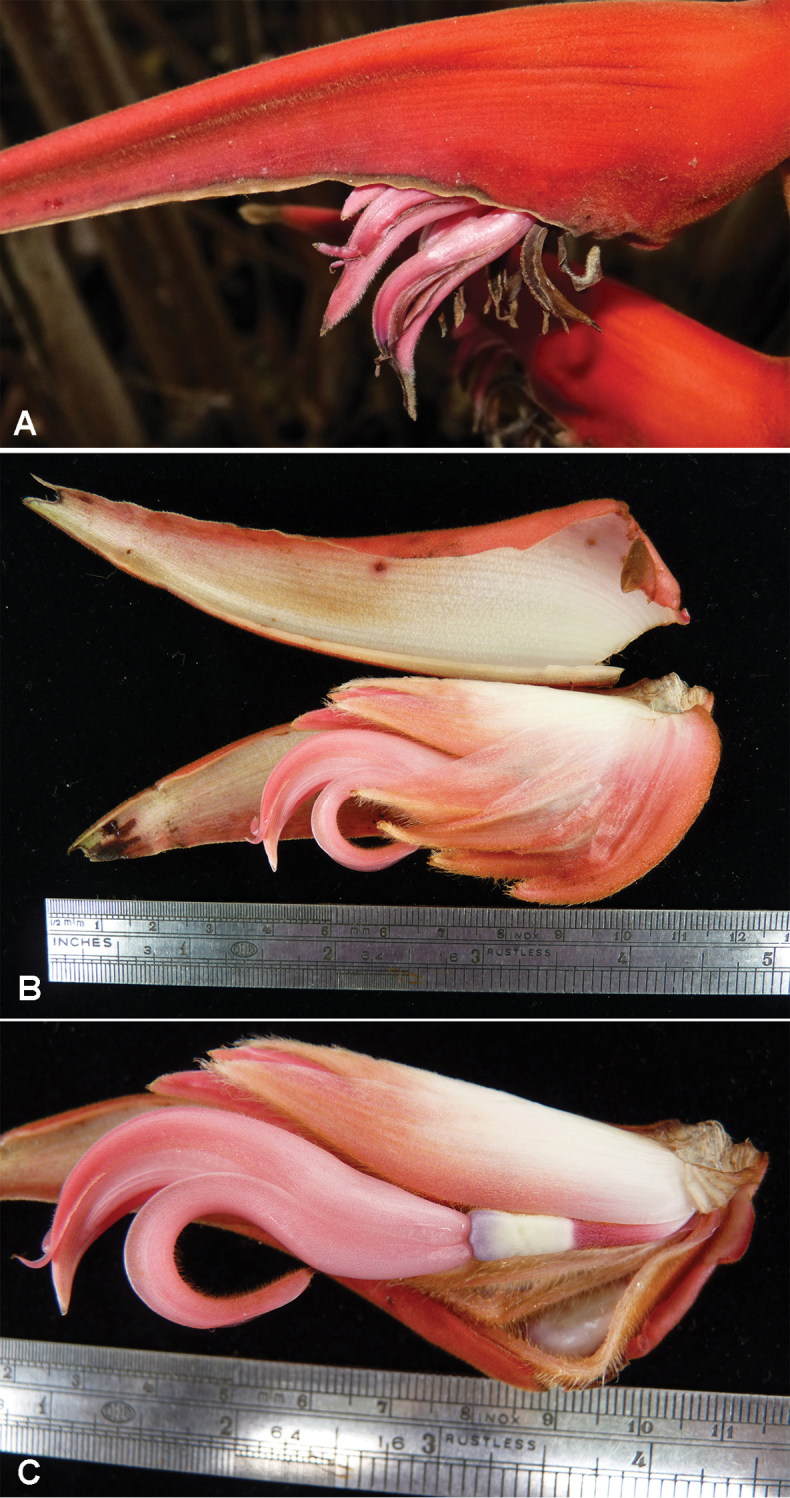
**A** Inflorescence segment of *Heliconia berguidoi* **B** Cincinnal bracts opened, showing floral bracts **C** Flower. Photos: **A** – R. Flores; **B, C** – C. Black.

**Figure 4.**
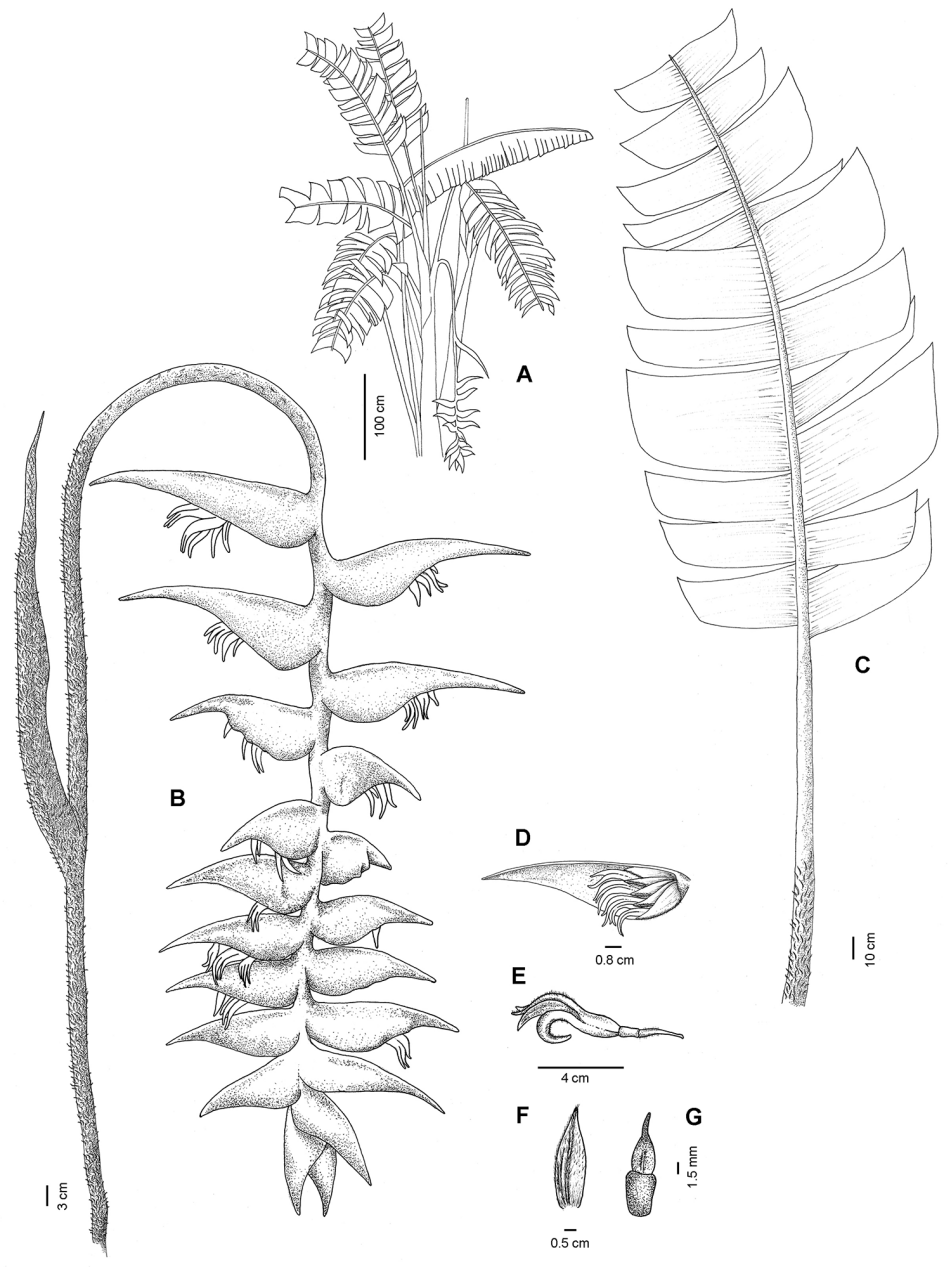
*Heliconia berguidoi* R.Flores, C.Black & A.Ibáñez. **A** Habit **B** Inflorescence **C** Leaf **D** Cincinnal bract open and flowers **E** Flower **F** Flower bract **G** Staminode.

Cincinnal bracts spirally arranged, ca. 25 per inflorescence, oriented ca. 120° to axis of the inflorescence, normally a sterile bract inserted in the peduncle, basal bracts separated ca. 3 cm and 1.5 cm between terminal bracts, the basal bract more elongated, outer surface pink at the base, turning red at the apex, totally velutinous with golden hairs, inner surface whitish, glabrous at the base with a few grouped hairs on both sides of the base, pink and hirsutulous with golden hairs at the margins and in the middle of the bract, ca. 12 cm long, ca. 5 cm wide at the base, l/w= 2.4 (Figures 3A, 4D).

Floral bracts persistent, 4.2–5.5 cm long, 1.5–2.6 cm wide at the base, pink, carinate, base of the abaxial surface glabrous to slightly tomentose at the apex, adaxial surface slightly tomentose at the base, inner surface glabrous (Figures 3B, 4F).

Flowers (5-)11–21 per cincinnus; pedicel pink, white at the base, pubescent, 12–20 mm long; ovary 10–11 mm long, 5 mm in diameter, lavender, glabrous; perianth 4.5–5.5 cm long, 0.6-0.8 cm in diameter, at anthesis curved 80° and sigmoid, slightly pink at the base, dark pink at the apex, glabrous except for pubescence at the apex of the perianth; free sepal reflexed, fused sepals with apices reflexed (Figures 3C, 4E); staminode 7.0–7.5 by 2.5–3.0 mm, white, fused to the perianth tube 12 mm above the base, elliptic with cuspidate apex (Figure 4G); stamens with anthers connivent and inside corolla apex. Drupes glabrous, bright blue 10–14 mm long, 9 mm wide.

#### Distribution, habitat and ecology.

*Heliconia berguidoi* is endemic to the Serranía de Majé, eastern Panama. It is known only from the Chucantí Private Nature Reserve (Figure 1), where it inhabits premontane forest at ca. 800 m. The canopy in this area attains a height of 20–35 m; some common canopy species are *Oreomunnea pterocarpa*, *Ocotea* sp. nov. ined., *Magnolia* sp. nov. ined., *Quercus humboldtii*, *Podocarpus guatemalensis* and *Peltogyne purpurea*. One population of *Heliconia berguidoi* has been found growing in early secondary forest regenerating from previous clearance or treefall gaps. It also appears in mature forest. Mature flowers were collected *in situ* in March, while *ex situ* the species seems to flower all year round. Mature fruits have not been collected *in situ*. *Ex situ* they appear all year round.

#### Conservation status.

*Heliconia berguidoi* is known only from one population in the type locality, Chucantí Private Nature Reserve. Human activities such as agriculture, cattle ranching and logging are the main threats to other populations of this species that probably exist in the forests around the reserve. These areas belong to farmers engaged in the activities mentioned above or else are public lands prone to colonization. Because of the restricted area of occupancy (AOO) estimated at 4 sq. km, and the severe threats, we consider that *Heliconia berguidoi* fits the category of Critically Endangered [CR B2ab (ii, iii, iv)] of the IUCN Red List and criteria (IUCN 2012).

#### Etymology.

The specific epithet, ***berguidoi***, honors the Panamanian biologist Guido Cesar Berguido F., who first brought national attention to Cerro Chucantí after witnessing not only its natural splendor, but the rampant ongoing deforestation. He mustered support from family and friends to purchase a property and set it aside for conservation before the previous owners could burn the forest to ashes. He received further private support and acquired more lands to create the Chucantí Private Nature Reserve. Mr. Berguido continues to invite fellow biologists to study the flora and fauna of Cerro Chucantí, which has resulted in the discovery of various species new to science. He recently founded the *Asociación Adopta el Bosque Panamá*, Adopt a Panama Rainforest, ADOPTA (www.chucanti.org) to further his conservation mission. It is an honor to thus recognize Mr. Berguido´s contributions to increased biological knowledge and his great efforts to conserve the unique forests of Cerro Chucantí. His generous logistical assistance to the authors was invaluable.

#### Paratypes.

PANAMÁ. Provincia de Darién. Cerro Chucantí, 800 m, 8°47'15.84"N, 78°27'13.57"W, 3 marzo 2011, fl., R. Flores & K. Morales. 595 RF (PMA!).

## Discussion

The new species of *Heliconia* described here belongs to the section Barbatae J.Kress ined., characterized by having the inflorescence, parts of it and/or the flowers densely pubescent with colored hairs (Kress et al. 1999, Kress and Betancur 2009) and to subgenus *Griggsia* L.Andersson characterized by a pendent inflorescence (Kress 1990a). It is the first species of both subgenus and section found in Panama with pink flowers and very long peduncle, which occasionally makes the inflorescence touch the ground. Very few species in the genus *Heliconia* have pink flowers.

It resembles the Colombian species *Heliconia rhodantha* and *Heliconia sanctae-theresae* in the pink flowers. Also, *Heliconia rhodantha* is similar to *Heliconia berguidoi* in the length and width of the leaf and the truncate and unequal leaf base. Nevertheless, the three species differ in several ways. *Heliconia berguidoi* has petioles up to 180 cm long with a woolly base vs. petioles that do not exceed 110 cm long and totally glabrous in *Heliconia rhodantha*, and up to 220 cm and totally glabrous in *Heliconia sanctae-theresae*. Inflorescences of *Heliconia berguidoi* reach 220 cm long with a wooly peduncle and a slightly flexuous, velutinous rachis; the cincinnal bracts are spirally arranged and velutinous with apex not early necrotic vs. inflorescences up to 135 cm long, pubescent, velvety peduncle and a flexuous, finely pubescent rachis; cincinnal bracts distichous and finely pubescent with apex early necrotic in *Heliconia rhodantha*, and inflorescences up to 67 cm long, velvety peduncle, flexuous rachis and cincinnal bracts distichous in *Heliconia sanctae-theresae*. The perianth of *Heliconia berguidoi* is pubescent at the apex and the elliptic staminode has a cuspidate apex vs. perianth glabrous and staminode completely linear in *Heliconia rhodantha*, and perianth finely pubescent and staminode ovolanceolate in *Heliconia sanctae-theresae*.

*Heliconia berguidoi* is similar to *Heliconia pogonantha* in its four varieties, mainly in the habit: leaves held more or less in horizontal position, leaves with unequal bases, blade splitting in segments, cincinnal bracts spirally arranged and the apex of the perianth pubescent. Additionally, it is similar to one of the varieties of *Heliconia pogonantha* (Heliconia pogonantha var. pubescens) in the combination of usually woolly peduncles and the rachises and cincinnal bracts velutinous.

*Heliconia berguidoi* differs clearly from *Heliconia pogonantha* in its four varieties by the petioles woolly towards the base, leaf with acuminate apex, long peduncle (125–150 cm), slightly flexuous rachis, pink flowers and staminode with cuspidate apex vs. petioles glabrous, leaf with acute apex, shorter peduncle (10–60 cm), flexuous rachis, yellow flowers and staminode with acuminate apex in *Heliconia pogonantha*. Additionally, the inflorescence is one-colored in *Heliconia berguidoi* vs. two-colored in three of the four varieties of *Heliconia pogonantha*.

With the combination of similar habit, blade splitting in segments, woolly peduncle, monochromatic cincinnal bracts and pubescent sepal apexes, *Heliconia berguidoi* is very close to two of the four varieties of *Heliconia ramonensis* (Heliconia ramonensis var. ramonensis and Heliconia ramonensis var. xanthotricha) but it is clearly differentiated by the longer peduncle (125–150 cm), slightly flexuous rachis and pink flowers vs. shorter peduncle (10–60 cm), flexuous rachis and yellow flowers.

With the description of *Heliconia berguidoi*, fifty-six native *Heliconia* species grow in Panama, eighteen of them endemic. This new species adds to a total of 178 species in the Neotropical region and 184 worldwide.

### Key to Panamanian Heliconia sect. Barbatae ined. with inclusion of *Heliconia rhodantha* and *Heliconia sanctae-theresae*, Colombian species. Based on Kress (1984) with modifications:

**Table:** 

1	Flowers pink, staminode totally linear, ovolanceolate or apex of staminode cuspidate.	
2	Peduncle absent or to up 14 cm, staminode ovolanceoate	***Heliconia sanctae-theresae***
2'	Peduncle 50–150 cm long, staminode linear or apex of staminode cuspidate.	
3	Perianth pubescent at the apex, staminode with the apex cuspidate	***Heliconia berguidoi***
3'	Perianth glabrous, staminode linear	***Heliconia rhodantha***
1'	Flowers yellow, apex of staminode acuminate or cuspidate	
4	Peduncle, rachis, and cincinnal bracts essentially glabrous.	
4'	Peduncle and rachis red or yellow, cincinnal bracts entirely red or red and yellow; floral bracts and perianth with golden hairs	***Heliconia pogonantha***
5	Peduncle, rachis, and cincinnal bracts rose-red; floral bracts and perianth with bright yellow hairs	***Heliconia ramonensis***
5'	Peduncle, rachis, and/or cincinnal bracts densely velutinous to woolly.	
6	Cincinnal bracts two-colored, red and yellow, velutinous	***Heliconia pogonantha***
6'	Cincinnal bracts one-colored, not red and yellow, velutinous to woolly.	
7	Inflorescence orange to rose-red, with orange hairs; perianth with orange or buff to rusty orange hairs.	
8	Inflorescence pink to rose-red, with rusty orange hairs; perianth with rusty orange hairs	***Heliconia ramonensis***
8'	Inflorescence orange-red with orange (fresh) or buff (dried) hairs	***Heliconia danielsiana***
7'	lnflorescence burgundy with golden to burgundy hairs or yellow with yellow hairs; perianth with golden or yellow hairs.	
9	Inflorescence deep red to burgundy, with golden to burgundy hairs; perianth with golden hairs	***Heliconia magnifica***
9'	Inflorescence bright yellow-green with yellow hairs; perianth with bright yellow hairs	***Heliconia xanthovillosa***

## Supplementary Material

XML Treatment for Heliconia berguidoi

## References

[B1] Andersson L (1985) Musaceae. In: Harling G Sparre B (Eds) Flora of Ecuador. Volume 22, 1–86.

[B2] Andersson L (1992) Revision of Heliconia subgen. Taeniostrobus and subgen. Heliconia (Musaceae-Heliconioideae). Opera Bot. 111: 1–98.

[B3] Angehr GR (2003) Directorio de áreas importantes para aves en Panamá. Directory of important bird areas in Panama. Sociedad Audubon de Panamá, Panamá.

[B4] APG IV (2016) An update of the Angiosperm Phylogeny Group classification for the orders and families of flowering plants: APG IV. Botanical Journal of the Linnean Society 181: 1–20. https://doi.org/10.1111/boj.12385

[B5] Batista A Köhler G Mebert K Vesely M (2014) A new species of *Bolitoglossa* (Amphibia: Plethodontidae) from eastern Panama, with comments on other species of the adspersa species group from eastern Panama. Mesoamerican Herpetology 1: 97–121.

[B6] Berry F Kress WJ (1991) *Heliconia*: An Identification Guide. Smithsonian Institution Press, 334 pp.

[B7] Bezark LG Tyson WH Schiff NM (2013) New species of Cerambycidae from Panama, with new distribution records (Coleoptera: Cerambycidae). Zootaxa 3608(4): 273–277. https://doi.org/10.11646/zootaxa.3608.4.5 24614469 10.11646/zootaxa.3608.4.5

[B8] BirdLife International (2016) Important Bird and Biodiversity Area factsheet: Serranía de Majé. http://www.birdlife.org [accessed 02/06/2016]

[B9] Correa MD Galdames C Stapf M (2004) Catálogo de Plantas Vasculares de Panamá. Smithsonian Tropical Research Institute, Panamá, 599 pp.

[B10] Ferreira de Castro CE May A Gonçalves C (2007) Atualização da nomenclatura de espécies do gênero *Heliconia* (Heliconiaceae). Revista Brasileira de Horticultura Ornamental 13(1): 38–62. https://doi.org/10.14295/rbho.v13i1.204

[B11] Holdridge LR Grenke WC Hatheway WH Liang T Tosi JAJ (1971) Forest environments tropical life zones. Pergamon Press, 747 pp.

[B12] IUCN (2012) Red List Categories and Criteria: Version 3.1. Second edition. IUCN Species Survival Commission. IUCN, Gland and Cambridge, 34 pp.

[B13] Kress WJ (1984) Systematics of Central American *Heliconia* (Heliconiaceae) with pendent inflorescenses. Journal of the Arnold Arboretum 65: 429–532.

[B14] Kress WJ (1990a) The diversity and distribution of *Heliconia* (Heliconiaceae) in Brazil. Acta Botanica Brasilica 4(1): 159–167. https://doi.org/10.1590/S0102-33061990000100011

[B15] Kress WJ (1990b) The taxonomy of old world *Heliconia* (Heliconiaceae). Allertonia 6: 1–58.

[B16] Kress WJ Betancur J Echeverry B (1999) Heliconias, Llamaradas de la Selva Colombiana. Guía de Campo. Cristina Uribe Editores Ltda., Primera Edición, Santafé de Bogotá, 200 pp.

[B17] Kress WJ Betancur J (2009) Una especie nueva de *Heliconia* (Heliconiaceae) del Chocó biogeográfico colombiano. Caldasia 31(1): 99–104.

[B18] Kress WJ (2003) Heliconiaceae In: Hammel BE Grayum MH Herrera C Zamora N (Eds) Manual de Plantas de Costa Rica (Vol. II): Gimnospermas y Monocotiledóneas (Agavaceae-Musaceae). Monographs in systematic botany from the Missouri Botanical Garden 92: 578–592.

[B19] Miranda RJ Bermúdez SE (2010) *Strophaeus sebastiani*, nueva especie de Barychelidae (Araneae: Mygalomorphae) de Panamá. Boletín de la Sociedad Entomológica Aragonesa (S.E.A.) 47: 175‒179.

[B20] Ortiz OO Baldini RM Berguido G Croat TB (2016) New species of *Anthurium* (Araceae) from Chucantí Nature Reserve, eastern Panama. Phytotaxa 255(1): 47–56. https://doi.org/10.11646/phytotaxa.255.1.4

[B21] TROPICOS (2016) Tropicos.org. Missouri Botanical Garden http://www.tropicos.org [accessed 7 Nov. 2016]

[B22] WWF (2016) Terrestrial Ecoregions. Tropical and subtropical broadleaved forests. Neotropical. Central America: Panama and Colombia. http://www.worldwildlife.org/ecoregions/nt0122

